# Prescription patterns of direct oral anticoagulants and concomitant use of interacting medications in the Netherlands

**DOI:** 10.1007/s12471-021-01612-4

**Published:** 2021-08-18

**Authors:** R. E. Harskamp, J. C. L. Himmelreich, G. W. M. Wong, M. Teichert

**Affiliations:** 1 Department of General Practice, Amsterdam UMC, University of Amsterdam, Academic Medical Center, Amsterdam, The Netherlands; 2 Department of Clinical Pharmacy and Toxicology, Leiden University Medical Centre, Leiden, The Netherlands

**Keywords:** Direct oral anticoagulant, Novel oral anticoagulant, Interactions, Community

## Abstract

**Objectives:**

To describe the prevalence, temporal and regional trends in prescribing direct oral anticoagulants (DOACs) in conjunction with interacting medications.

**Methods:**

We performed a cross-sectional study of pharmacy dispensing data in the Foundation for Pharmaceutical Statistics (SFK) registry on patients who have had a prescription for a DOAC filled at one of 831 randomly selected pharmacies in the Netherlands between Jan 2014–Jan 2019.

**Results:**

We identified 99,211 patients who had a first DOAC prescription filled. Mean age was 71.6 ± 10.9 years, 58% were male. In 2014, 8,293 patients were treated with DOACs, in 2018, 35,415 were newly started on a DOAC. In 2018, the use of apixaban was most common (52%) in the Eastern region, whereas rivaroxaban was most frequently prescribed (32–48%) in the other regions. At time of first prescription, the vast majority (99.3%) used ≥ 1 concomitant interacting drug, and 3.2% used ≥ 3 interacting medications. Most common were digoxin (37.8%), atorvastatin (31.5%), verapamil (13.7%) and amiodarone (9.7%). While the number of interacting medications remained unchanged over time (median 1, interquartile range 1–1), there was a notable decrease in antiarrhythmic medications and an increase in non-cardiovascular interacting medications (e.g. dexamethasone from 0.9% to 7.1%, antiepileptic drugs from 2.5% to 3.8%, and haloperidol from 0.5% to 2.2% in 2014 and 2018, respectively).

**Conclusion:**

DOAC use has quadrupled in Dutch clinical practice over the 5‑year period from 2014 to 2018. While the number of patients who take interacting medications remained stable, the profile of interacting medications has changed over time from cardiovascular to medications affecting other organ systems.

**Supplementary Information:**

The online version of this article (10.1007/s12471-021-01612-4) contains supplementary material, which is available to authorized users.

## What’s new?

- The use of direct oral anticoagulants (DOACs) has quadrupled in Dutch clinical practice over a 5-year period in the late 2010s.
- There is considerable regional variation in which specific DOAC is preferred.
- The number of patients who take interacting medications has also quadrupled.
- The profile of interacting medications has changed over time from cardiovascular to medications affecting other organ systems, suggesting DOAC use is expanded to more diverse patient populations.
- This manuscript includes a chart with common DOAC-drug interactions and refers to easy-to-use drug interaction tools.

## Introduction

Direct oral anticoagulants (DOACs) have been developed as an alternative for vitamin K antagonists (VKAs) [1]. The main indication is atrial fibrillation (AF), in which DOACs have demonstrated comparable efficacy in preventing thromboembolic stroke and lower bleeding risk [2]. DOACs have been successfully implemented in clinical care and have overtaken VKAs as the preferred oral anticoagulant in the late 2010s [3]. However, despite DOACs being marketed as “hassle free”, physicians must be aware of drug-drug interactions of DOACs [4]. The European Heart Rhythm Association (EHRA) developed a DOAC prescription guide for patients with AF [5]. The EHRA guide lists a number of medications that cause drug-drug interactions, in which DOACs should be used with caution, or perhaps better avoided. In the current study, we aimed to describe the uptake of DOACs in the Netherlands over a 5-year period in the late 2010s in relation to the absolute and relative use of concomitant medications that are listed as causing drug-drug interactions.

## Methods

For the current study we adhered to the “Reporting of studies conducted using observational routinely-collected health data (RECORD) statement”[6].

### Study design and setting

Our study involved a cross-sectional study for the calendar years 2014 up to and including 2018 of pharmacy dispensing data in patients who had their prescriptions for DOACs filled in a community pharmacy connected to the Dutch Foundation for Pharmaceutical Statistics (SFK). The SFK obtains data from > 95% of the community pharmacies in the Netherlands and covers about 15.8 million people (on a population of 17.3 million) (https://www.sfk.nl). Information on patients’ sex and year of birth were available. Dispensing data were collected within pharmacies on a patient level, in which each dispensing was linked to the individual’s unique identification number. For each dispensation, we also obtained information about the drug supplied, and the postal code (two first numbers to indicate a postal region) of the dispensing pharmacy. Dispensations for one person could not be merged from different pharmacies. Drug dispensing medication was available as Anatomical Therapeutic Chemical (ATC) codes. Privacy of participating pharmacists, as well as prescribing doctors and patients, was guaranteed by providing the investigators with restricted access to a de-identified data file.

### Participants and prescription data

We only included community pharmacies that provided complete data over a 5-year time window and did not change software (which involved 1,665 out of 1,981 pharmacies). From these pharmacies we randomly selected a representative sample (approximately half of these pharmacies), as we deemed this number sufficient to address our research question. We obtained annual prescription data of patients who had at least one prescription for DOACs filled between 1 January, 2014, and 1 January, 2019. The DOACs we included were dabigatran, rivaroxaban, apixaban, and edoxaban. For each dispensation, we also obtained information about the DOAC and interacting drugs supplied. First dispensations of DOACs were defined as a DOAC dispensation without a dispensation of any DOAC in the previous 12 months and a dispensing fee for a first dispensing registered by the pharmacy.

### Outcomes of interest

The outcome of interest was concomitant use of a DOAC and one or more of the selected medications with a known drug-drug interaction profile as listed in the EHRA document [5] complemented with psychotropic agents known to cause drug-drug interactions [7]. A list of the medications, as well as the presumed interaction mechanism for each DOAC can be found in supplementary Table S1. We defined concomitant use when there was a 30-day overlap between a DOAC and another drug, except for medications that are typically used for shorter durations (i.e. macrolides), for which we used a 5-day overlap. We recorded the concomitant use of interacting medications for each calendar year.

### Statistical analyses

Data were presented using the total number of prevalent DOAC users within a study year as well as new users of DOACs. We displayed categorical variables as median (interquartile range—IQR) or as percentage and number in parentheses, and compared these using the chi-square or Fisher’s exact test. For continuous variables we used mean ± standard deviation or median (IQR) quartiles depending on whether there was a normal distribution (based on skewness and kurtosis). We compared continuous data using the independent samples t‑test for normally distributed variables, and the Mann-Whitney U test for non-normally distributed variables.

## Results

### Participants

In 831 community pharmacies there were 179,748 DOAC prescriptions to 99,211 unique patients within the separate pharmacies over the 5‑year study period. The total number of DOAC prescriptions rose from 8,293 in the calendar year 2014 to 74,461 in 2018. Over time, age as well as proportion of females among first-time DOAC users increased significantly, from mean age 70.6 ± 9.9 years and 39% female in 2014, to mean age 72.2 ± 11.1 years and 42% female in 2018 (see Fig. S1 in Electronic Supplementary Material [ESM]).

### Trends among new DOAC users

**Fig. 1:**
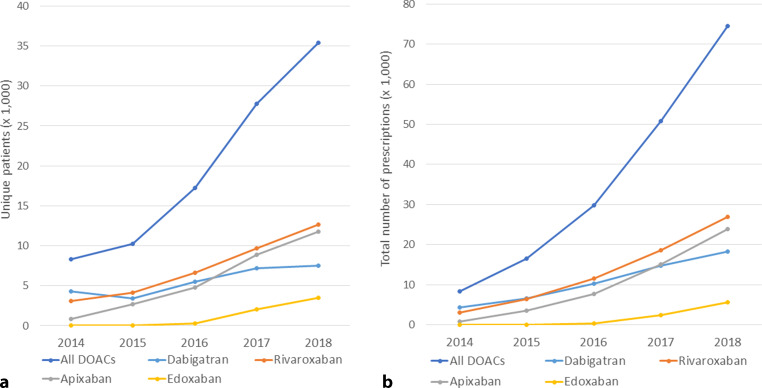
Temporal trends in DOAC use in the Netherlands (2014–2018).** a** Number of patients with a first DOAC dispensation (per year); **b** Total number of DOAC dispensations (per year). *DOAC* direct oral anticoagulant

**Fig. 2:**
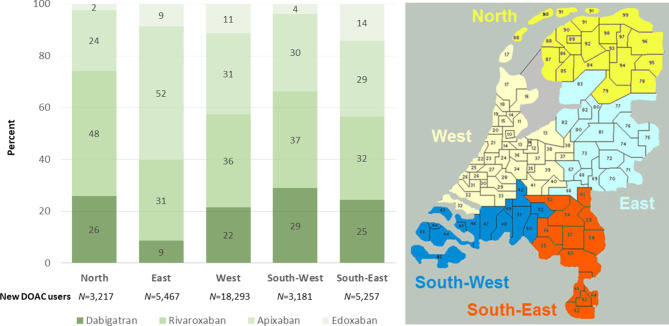
Regional variation in DOAC prescription among first time users in 2018. Displayed are the share of total patients with a first DOAC dispensation in 2018 in five different regions in the sample (*left panel*), and the geographic confinements of each of the five regions referred to in the left panel (*right panel*). *DOAC* direct oral anticoagulant

The initiation of DOAC treatment quadrupled over a 5-year time window. In our sample of 831 pharmacies, 8,293 patients were given their first DOAC prescription in 2014, while in 2018 a total of 35,415 were newly started on a DOAC. Temporal trends in the use of individual DOACs are displayed in Fig. 1. All DOACs showed an increase in use over time. This increase appeared to go in parallel for individual DOACs, except for dabigatran. Overall, rivaroxaban was most frequently prescribed (*n* = 12,672) among new DOAC users, followed by apixaban (*n* = 11,784), dabigatran (*n* = 7,473) and lastly edoxaban (3,486). The uptake of DOACs as a group was comparable between five geographical regions of the Netherlands over time, however there were notable differences in the preferred type of DOAC. As shown in Fig. 2*,* apixaban (52%) was most commonly prescribed to new DOAC users in the Eastern region of the Netherlands in 2018, whereas rivaroxaban was most frequently prescribed (32–48%) in the other regions.

### Trends in use of concomitant interacting medications

**Fig. 3:**
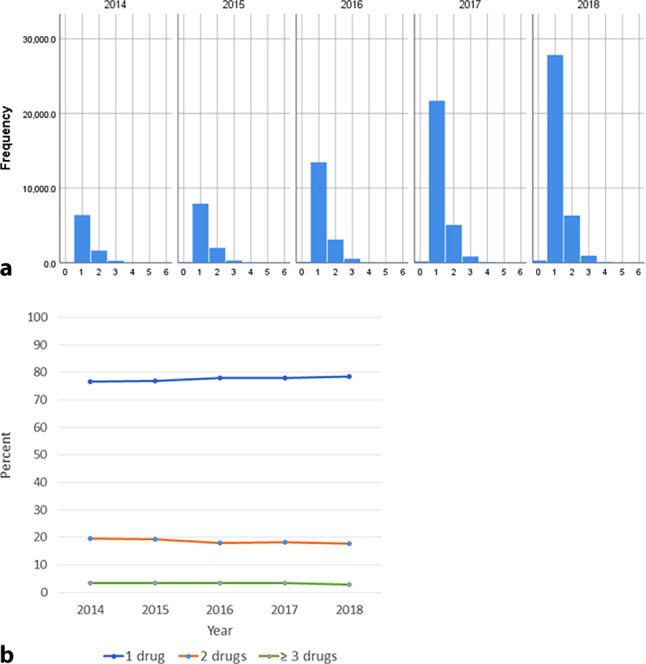
Temporal trends in use of DOAC-interacting medication (2014–2018). **a** Histograms showing the number of patients with 0, 1, 2, 3, 4, 5 or 6 DOAC-interacting drugs at first DOAC dispensation over the study years; **b** Patients in the sample with 1 (*blue*), 2 (*orange*), or ≥ 3 (*green*) interacting drugs at first DOAC dispensation over the study period as percentage of total number of new DOAC users in each reference year. *DOAC* direct oral anticoagulant. **a** Number of DOAC-interacting drugs per calender year. **b** New users with DOAC-interacting drugs (percentage)

The vast majority (99.3%) of included patients used concomitant interacting medications at time of first DOAC dispensation. Of those, most used either one (77.9%) or two (18.2%) interacting medications, with 4.8% using 3 or more. The most commonly prescribed interacting medications were digoxin (37.8%), atorvastatin (31.5%), verapamil (13.7%) and amiodarone (9.7%). As shown in Fig. 3, the distribution of the number of interacting drugs at first DOAC dispensation remained stable across the study years, as was the number of patients with 1, 2, or ≥ 3 interacting medications as percentage of total prescriptions. The median number of interacting medication remained 1 (IQR 1–1) across study years.

When assessing individual medications, we found a decrease in the use of antiarrhythmic medications between 2014 and 2018, most notably digoxin (41.7% versus 35.5%) and verapamil (20.5% versus 10.8%) (Tab. 1). On the other hand, there was a significant increase in this same period for concomitant use of many other interacting medications. Notable examples are atorvastatin (29.2% versus 31.9%), dexamethasone (0.9% versus 7.1%), amitriptyline (4.0% versus 5.3%), antiepileptic drugs (2.5% versus 3.8%), and haloperidol (0.5% versus 2.2%). As shown, in Tab. 2 these temporal changes can be observed for dabigatran, rivaroxaban as well as apixaban. In the latest DOAC prescription data (2018), differences were minimal between the individual DOACs, except for dexamethasone, which was more common among edoxaban users (8.8% versus 3.1–5.4% for the other DOACs).

**Table 1:** Use of interacting medications among new DOAC users per calendar year

	2014 (*n* = 8,345)	2015 (*n* = 10,292)	2016 (*n* = 17,249)	2017 (*n* = 27,857)	2018 (*n* = 35,468)	*P*-value
Amiodarone	12.6% (1,052)	11.6% (1,197)	10.4% (1,802)	9.2% (2,565)	8.5% (3,023)	< 0.001
Digoxin	41.7% (3,481)	40.3% (4,146)	38.9% (6,704)	38.0% (10,585)	35.5% (12,606)	< 0.001
Diltiazem	4.7% (395)	4.3% (439)	3.4% (584)	3.6% (990)	3.1% (1,107)	< 0.001
Quinidine	0.0% (1)	0.0% (0)	0.0% (2)	0.0% (5)	0.0% (5)	0.75
Verapamil	20.5% (1,712)	18.0% (1,857)	15.4% (2,652)	12.7% (3,532)	10.8% (3,822)	< 0.001
Atorvastatin	29.2% (2,438)	30.8% (3,169)	31.6% (5,446)	32.0% (8,913)	31.9% (11,305)	< 0.001
Clarithromycin	0.9% (75)	1.6% (161)	1.7% (291)	2.0% (547)	2.1% (738)	< 0.001
Erythromycin	0.3% (21)	0.2% (18)	0.1% (25)	0.2% (46)	0.3% (58)	0.40
Rifampicin (rifampin)	0.1% (7)	0.1% (8)	0.1% (21)	0.1% (24)	0.1% (50)	0.19
Protease inhibitors	0.0% (1)	0.0% (0)	0.0% (0)	0.0% (4)	0.0% (17)	0.001
Fluconazole	0.7% (58)	0.8% (79)	1.3% (227)	1.4% (399)	1.8% (642)	< 0.001
Keto/itraconazole	0.2% (18)	0.3% (32)	0.3% (41)	0.2% (65)	0.3% (88)	0.68
Carbamazepine	0.7% (55)	0.4% (45)	0.4% (72)	0.5% (145)	0.4% (155)	0.04
Antiepileptic drugs	2.5% (205)	2.6% (268)	3.0% (526)	3.4% (958)	3.8% (1,334)	< 0.001
Antimitotic drugs	0.0% (0)	0.0% (0)	0.0% (0)	0.0% (3)	0.1% (30)	< 0.001
Doxorubicin	0.0% (0)	0.0% (0)	0.0% (1)	0.0% (3)	0.1% (18)	< 0.001
Tyrosine kinase inhibitors	0.0% (0)	0.1% (12)	0.1% (11)	0.1% (38)	0.2% (73)	< 0.001
Antihormonal drugs	0.8% (66)	1.1% (114)	1.2% (199)	1.4% (383)	1.7% (589)	< 0.001
Calcineurin inhibitors	0.1% (7)	0.2% (19)	0.3% (60)	0.7% (193)	0.9% (334)	< 0.001
Dexamethasone	0.9% (78)	2.1% (215)	3.3% (573)	4.6% (1,285)	7.1% (2,519)	< 0.001
Sertraline	1.0% (84)	1.0% (108)	1.5% (255)	1.6% (444)	1.4% (496)	< 0.001
Venlafaxine	2.1% (175)	2.1% (217)	2.2% (387)	2.2% (618)	2.1% (742)	0.72
Amitriptyline	4.0% (322)	4.1% (418)	5.0% (863)	5.3% (1,479)	5.3% (1,895)	< 0.001
Haloperidol	0.5% (44)	0.7% (76)	1.0% (168)	1.6% (441)	2.2% (786)	< 0.001
Methotrexate	2.8% (235)	3.2% (333)	3.3% (563)	3.6% (996)	3.3% (1,188)	0.01

**Table 2:** Use of concomitant medications at first direct oral anticoagulant (DOAC) dispensation with significant temporal changes between 2014 and 2018, presented per DOAC

	Dabigatran		Rivaroxaban		Apixaban		Edoxaban	
	2014 (*n* = 4,324)	2018 (*n* = 7,473)	2014 (*n* = 3,103)	2018 (*n* = 12,672)	2014 (*n* = 866)	2018 (*n* = 11,784)	2014 (*n* = 0)	2018 (*n* = 3,486)
Number of interacting drugs	1.27 ± 0.53	1.23 ± 0.51	1.26 ± 0.54	1.21 ± 0.50	1.24 ± 0.49	1.23 ± 0.51	–	1.28 ± 0.56
Amiodarone	12.8% (554)	10.8% (1969)	12.4% (385)	8.9% (2,389)	11.8% (102)	9.9% (2,368)	–	10.6% (591)
Digoxin	42.9% (1,855)	38.9% (7,083)	39.3% (1,221)	35.5% (9,547)	43.2% (374)	40.3% (9,598)	–	37.1% (2,063)
Diltiazem	4.9% (212)	3.7% (683)	4.8% (149)	3.5% (937)	3.9% (34)	2.8% (655)	–	4.0% (222)
Verapamil	19.2% (829)	14.0% (2557)	22.7% (704)	13.7% (3676)	19.7% (171)	12.6% (3,009)	–	12.2% (677)
Atorvastatin	29.8% (1,287)	35.9% (6,534)	28.9% (897)	34.0% (9,125)	28.1% (243)	34.9% (8,307)	–	32.8% (1,825)
Clarithromycin	1.1% (47)	1.8% (324)	0.6% (18)	1.2% (313)	0.9% (8)	1.2% (294)	–	1.3% (75)
Protease inhibitors	0.0% (1)	0.0% (9)	0.0% (0)	0.0% (6)	0.0% (0)	0.0% (2)	–	0.1% (4)
Fluconazole	0.9% (37)	1.1% (204)	0.5% (16)	1.3% (341)	0.5% (4)	1.3% (306)	–	1.6% (90)
Antiepileptic drugs	2.6% (114)	3.6% (665)	2.3% (70)	3.9% (1,042)	2.3% (20)	3.8% (898)	–	3.4% (188)
Antimitotic drugs	0.0% (0)	0.0% (3)	0.0% (0)	0.1% (19)	0.0% (0)	0.0% (9)	–	0.1% (4)
Doxorubicin	0.0% (0)	0.0% (3)	0.0% (0)	0.1% (15)	0.0% (0)	0.0% (0)	–	0.1% (3)
Tyrosine kinase inhibitors	0.0% (0)	0.2% (29)	0.0% (0)	0.2% (45)	0.0% (0)	0.1% (26)	–	0.2% (13)
Antihormonal drugs	0.7% (32)	1.1% (197)	1.0% (30)	1.7% (462)	0.3% (3)	1.2% (276)	–	2.1% (115)
Calcineurin inhibitors	0.1% (3)	0.1% (22)	0.1% (2)	0.8% (215)	0.2% (2)	1.0% (242)	–	0.8% (47)
Dexamethasone	0.9% (41)	3.1% (566)	1.0% (31)	5.4% (1462)	0.7% (6)	3.8% (912)	–	8.8% (491)
Sertraline	0.9% (41)	1.3% (240)	1.3% (40)	1.7% (455)	0.3% (3)	1.5% (348)	–	1.5% (79)
Amitriptyline	3.8% (163)	4.6% (837)	3.8% (118)	5.9% (1,572)	5.8 (50)	5.0% (1,192)	–	4.8% (266)
Haloperidol	0.6% (25)	1.6% (299)	0.5% (16)	1.5% (391)	0.3% (3)	1.7% (395)	–	1.6% (87)
Methotrexate	2.5% (108)	3.2% (588)	3.4% (105)	3.9% (1,061)	2.4% (21)	3.5% (834)	–	3.4% (187)

Data are mean ± standard deviation and percentage (number).

## Discussion

In this representative sample of community pharmacies in the Netherlands, we found that the number of new DOAC patients as well as total number of DOAC dispensations quadrupled between 2014 and 2018. The median number of interacting drugs used at first DOAC dispensation remained stable over time, while the type of interacting drug changed from cardiovascular medications towards medications affecting other organ systems, as well as immune-modulating agents.

Increasing rates of DOAC prescription have been described in a number of prior reports from Europe and North America [8–14]. These studies also indicated that this did not come fully at the expense of VKA. Instead, vulnerable patients who were previously not receiving anticoagulation, are now receiving DOACs, as they are deemed potentially safer than VKAs, particularly in vulnerable patients at high risk of bleeding [8]. Prior studies also suggest that the patient profile associated with DOAC initiation has changed over time [8]. Among general practitioners, for instance, DOACs were initially prescribed with greater caution owing to preliminary uncertainties with regard to their safety and efficacy in primary care [4, 8, 15]. While our study did not collect in-depth patient characteristics, we did find that the type of interacting drugs changed over time. We also found that age at time of first DOAC dispensation increased over time. These changes likely indicate that the case mix of new and experienced DOAC users is changing, presumably because in the Netherlands general practitioners are also allowed to prescribe DOACs since the end of 2016. A possible explanation is that physicians have become more confident in using DOACs in higher risk patients with increased experience. Finally, this could also be attributed to pharmaceutical marketing effects, such as for edoxaban, which is being marketed for more vulnerable patient populations.

When assessing individual DOACs, other studies also found that dabigatran had a slower uptake, and was eventually overtaken by rivaroxaban [8, 9, 12], or in some countries by rivaroxaban and apixaban [16]. One possible explanation for dabigatran’s lack of continued growth lies in its mechanism of action and pharmacological characteristics which differs from the other DOACs. Dabigatran has a longer half-life and is primarily cleared renally [17]. This may make physicians more hesitant to prescribe this DOAC as it increases the risk of overdose, particularly in the presence of renal impairment [8]. From our data, however, we were unable to draw conclusions as to the reason for the observed trend in dabigatran uptake.

### Drug-drug interactions and clinical outcomes

Studies that assessed the impact of drug-drug interaction on clinical outcomes among DOAC users are limited. We have provided a summary of these studies in Tables S1 and S2 in Electronic Supplementary Material [ESM] [18–20]. To date, the study with the most impact is a Taiwanese population-based analysis, in which the authors found substantially increased bleeding risk among DOAC users who also used amiodarone, fluconazole, rifampicin (rifampin), and phenytoin [19]. Of interest, both the use of fluconazole and antiepileptic drugs have increased over time in our analysis. The Taiwanese study also found that other drugs which shared key metabolic pathways with DOACs that would mechanistically result in an increased bleeding risk, did not do so. The most intriguing example is the concomitant use of atorvastatin, which was associated with a lower risk of bleeding. In our study almost one-third of the population was using this type of statin. In contrast to atorvastatin, other statins such as simvastatin and lovastatin were associated with higher bleeding risk when used concomitantly with dabigatran in a study by Antoniou *et al*. [18]. These findings underline that pharmacokinetic data may not always line up with associations observed in clinical outcome-based studies. Unfortunately, the Taiwanese study does not provide information on the impact on efficacy outcomes, such as stroke and mortality, nor on the relative efficacy and safety when compared with warfarin. A recent meta-analysis based on post hoc analyses from randomised controlled trials seems to provide reassuring answers in that regard, as the data clearly demonstrate that in the presence of interacting drugs, the use of DOACs remains more effective than warfarin [21].

### Clinical guidance

So what are the clinical implications of our study? First, physicians should be aware that while DOACs may have fewer interactions than do VKAs, they do exist, and could lead to serious complications. Prior studies indicate that clinicians recognise only half of common drug-drug interactions as well as disease contraindication pairs [22]. Therefore, an improvement in the clinician’s ability to detect possible interactions could improve patients’ safety. We would therefore recommend that physicians who manage patients with AF use an online guidance tool, such as https://www.NOACforAF.eu, when considering DOAC therapy or adding a different treatment among DOAC users. On the mentioned website, physicians can find practical information on prescribing DOACs, which is based on the EHRA practical guide, along with patient information in different languages (including Dutch). There are also other sources to help check for possible drug-drug interactions, such as Lexicomp® (integrated in UpToDate), or the tool by Medscape, which can be directly assessed via https://reference.medscape.com/drug-interactionchecker [23].

### Limitations

Our study relied on prescription data from a representative sample of community-based pharmacies in the Netherlands, in which we presume that these medications had been filled and had been used by the patients. The database did not collect in-depth data on patient characteristics, indication for DOAC use, nor on clinical outcomes while using the DOAC, which did not allow us to draw inferences on proper dosing, or on the impact of interacting drugs on the risks of bleeding and stroke/systemic embolism. Moreover, the SFK database did not allow for merging individual subjects’ data in the case of moving to a different SFK pharmacy. This may have led to overestimation of the number of first DOAC users in the observed period. However, this is unlikely to bias the comparison between the study years and overestimation may be limited as shopping behaviour is typically low in older patients.

## Conclusion

The use of DOACs has quadrupled in Dutch clinical practice over the 5‑year period from 2014 to 2018. The rate of concomitant interacting medication remained stable over time. The profile of interacting medications has changed over time from cardiovascular to medications affecting other organ systems. Continued monitoring is warranted now that DOACs have expanded to patient populations that used medications whose interacting profiles were understudied in randomised controlled trials.

## Supplementary Information

Supplementary Table S1 Effect of drug-drug interactions on DOAC plasma levels, and recommendations for concomitant use with DOAC as per EHRA Guidelines, of the drugs included in the current analysis. Supplementary Table S2 Studies on drug-drug interactions with data on clinical outcomes

**Supplementary Fig. S1. **Mean age and percentage female and male among new DOAC users (2014–2018). Left panel: displayed are mean age (*points*) and 95% confidence intervals (*brackets*) per study year. Right panel: percentage female (*orange bars*) and male (*blue bars*) per study year. *DOAC* direct oral anticoagulant
