# Supplementary material for: Prescription patterns of direct oral anticoagulants and concomitant use of interacting medications in the Netherlands
Source: Neth Heart J. 2021 Aug 18;29(9):451–9. doi: 10.1007/s12471-021-01612-4 (PMC8397808; doi:10.1007/s12471-021-01612-4)
Supplement: Supplementary file 1 — Supplementary Table S1 Effect of drug-drug interactions on DOAC plasma levels, and recommendations for concomitant use with DOAC as per EHRA Guidelines, of the drugs included in the current analysis. Supplementary Table S2 Studies on drug-drug interactions with data on clinical outcomes [file 12471_2021_1612_MOESM1_ESM.docx]

Supplementary data to Harskamp et al., “Prescription patterns of direct oral anticoagulants and concomitant use of interacting medications in the Netherlands”

Supplementary Tables

**Supplementary Table S1** Effect of drug-drug interactions on DOAC plasma levels, and recommendations for concomitant use with DOAC as per EHRA Guidelines, of the drugs included in the current analysis

|  | **Drug** | **ATC** | **Pathway** | **Dabigatran** | **Apixaban** | **Edoxaban** | **Rivaroxaban** |
| --- | --- | --- | --- | --- | --- | --- | --- |
| Cardiovascular | amiodarone | C01BD01 | P-gp |  |  |  |  |
|  | digoxin | C01AA05  C01AA02 | P-gp | No direct effect | No direct effect | No direct effect | No direct effect |
|  | diltiazem | C05AE03  C08DB01 | P-gp, CYP3A4 | No effect |  | No data | No effect |
|  | quinidine | C01BA01  C01BA13 | P-gp |  | No data |  |  |
|  | verapamil | C08DA01  C08DA51 | P-gp, CYP3A4 |  | No data |  | No effect |
|  | atorvastatin | C10AA05 | P-gp, CYP3A4 | No effect | No data | No effect | No effect |
| Antibiotics | clarithromycin | J01FA09 | P-gp, CYP3A4 |  |  |  |  |
|  | erythromycin | J01FA01 | P-gp, CYP3A4 |  |  |  |  |
|  | rifampicin (rifampin) | J04AB02 | P-gp, BCRP, CYP3A4/CYP2J2 |  |  |  |  |
| Antiviral | protease inhibitors | J05AE03  J05AE08 J05AE10 | P-gp, BCRP, CYP3A4 |  |  |  |  |
| Fungostatics | fluconazole | J02AC01 | CYP3A4 | No data | No data | No data |  |
|  | ketoconazole, itraconazole | J02AB02  J02AC02 | P-gp, BCRP, CYP3A4 |  |  |  |  |
| Epileptic drugs | carbamazepine | N03AF01 | P-gp, CYP3A4 |  |  |  |  |
|  | levetiracetam, phenobarbital, phenytoin, topiramate, valproic acid | N03AX14  N03AA02  N03AB02  N03AX11  N03AG01 | P-gp, CYP3A4 |  |  |  |  |
| Antimitotic agents | paclitaxel  vinblastine | L01CD01  L01CA01 | P-gp, CYP3A4 |  |  |  |  |
| Anthracyclines | doxorubicin | L01DB01 | P-gp, CYP3A4 |  |  |  |  |
| Tyrosine kinase inhibitor | imatinib, crizotinib, nilotinib, lapatinib, vandetanib, sunitinib | L01XE01  L01XE16  L01XE08  L01XE07  L01XE12  L01XE04 | P-gp, CYP3A4 |  |  |  |  |
| Hormonal agents | abiraterone, enzalutamide, tamoxifen | L02BX03  L02BB04  L02BA01 | P-gp, CYP3A4 |  |  |  |  |
| Immune-modulating agents | calcineurin inhibitors: ciclosporin, tacrolimus | L04AD01  L04AD02 | P-gp, CYP3A4 |  |  |  |  |
|  | dexamethasone | H02AB02 | CYP3A4 |  |  |  |  |
| Psychotropic agents | sertraline | N06AB06 | No data | No data | No data | No data | No data |
|  | venlafaxine | N06AX16 | No data | No data | No data | No data | No data |
|  | amitriptyline | N06AA09 | No data | No data | No data | No data | No data |
|  | haloperidol | N05AD01 | No data | No data | No data | No data | No data |
| Antimetabolites | methotrexate | L04AX03 | P-gp | No data | No data | No data | No data |

Effect of drug-drug interactions on DOAC plasma levels as referenced in the 2018 European Heart Rhythm Association Practical Guide on the use of non-vitamin K antagonist oral anticoagulants in patients with atrial fibrillation [5], unless stated otherwise.

Yellow: use caution, consider dose adjustment or different DOAC; Dark Red: contraindicated/not recommended (increased DOAC plasma levels); Light blue: use caution or avoid (decreases DOAC plasma level); Dark blue: contraindicated (marked reduction of DOAC plasma levels).

*DOAC* direct oral anticoagulant, *EHRA* European Heart Rhythm Association, *ATC* anatomical therapeutic chemical

**Supplementary Table S2** Studies on drug-drug interactions with data on clinical outcomes

|  | ***N*** | **DOAC** | **Interacting drug** | **Outcome** | **OR/HR (95% CI)** | ***P*-value** |
| --- | --- | --- | --- | --- | --- | --- |
| Antoniou et al (18) | 45,991 | Dabigatran | Simvastatin/Lovastatin Simvastatin Lovastatin | Major haemorrhage | 1.46 (1.17-1.82)  1.44 (1.14-1.81)  1.90 (0.78-4.61) | NA^1^ NA NA |
| Chang et | 91,330 | Dabigatran | Amiodarone | Major bleeding risk | 1.36 (1.17-1.59)^*^ | <0.01 |
| al (19) |  | Rivaroxaban |  |  | 1.38 (1.21-1.58)^*^ | <0.01 |
|  |  | Apixaban |  |  | 1.30 (0.98-1.72)^*^ | NA |
|  |  | Dabigatran | Fluconazole | Major bleeding risk | 2.26 (1.44-3.55)^*^ | <0.01 |
|  |  | Rivaroxaban |  |  | 2.25 (1.54-3.30)^*^ | <0.01 |
|  |  | Apixaban |  |  | 3.36 (1.69-6.68)^*^ | <0.01 |
|  |  | Dabigatran | Rifampin | Major bleeding risk | 1.76 (0.91-3.42)^*^ | NA |
|  |  | Rivaroxaban |  |  | 1.59 (0.82-3.09)^*^ | NA |
|  |  | Apixaban |  |  | 0.49 (0.04-6.53)^*^ | NA |
|  |  | Dabigatran | Phenytoin | Major bleeding risk | 2.09 (1.53-2.85)^*^ | <0.01 |
|  |  | Rivaroxaban |  |  | 1.85 (1.36-2.51)^*^ | <0.01 |
|  |  | Apixaban |  |  | 1.80 (0.90-3.60)^*^ | NA |
| Kent et | 2,279 | Dabigatran | NSAID | Major bleeding | 1.68 (1.40-2.02) | <0.0001 |
| al (20) |  |  |  | GI major bleeding^2^ | 1.81 (1.35-2.43) | <0.0001 |
|  |  |  |  | Stroke or SE^3^ | 1.50 (1.12-2.01) | 0.007 |
|  |  |  |  | Hospitalisation | 1.64 (1.51-1.77) | <0.0001 |

^*^AIRR= Adjusted incidence rate ratio with 99% confidence interval

^1^NA= Not available

^2^GI= Gastro-intestinal bleeding

^3^SE= Systemic embolism

1
