# Supplementary figures and images for: Prescription patterns of direct oral anticoagulants and concomitant use of interacting medications in the Netherlands
Source: Neth Heart J. 2021 Aug 18;29(9):451–9. doi: 10.1007/s12471-021-01612-4 (PMC8397808; doi:10.1007/s12471-021-01612-4)

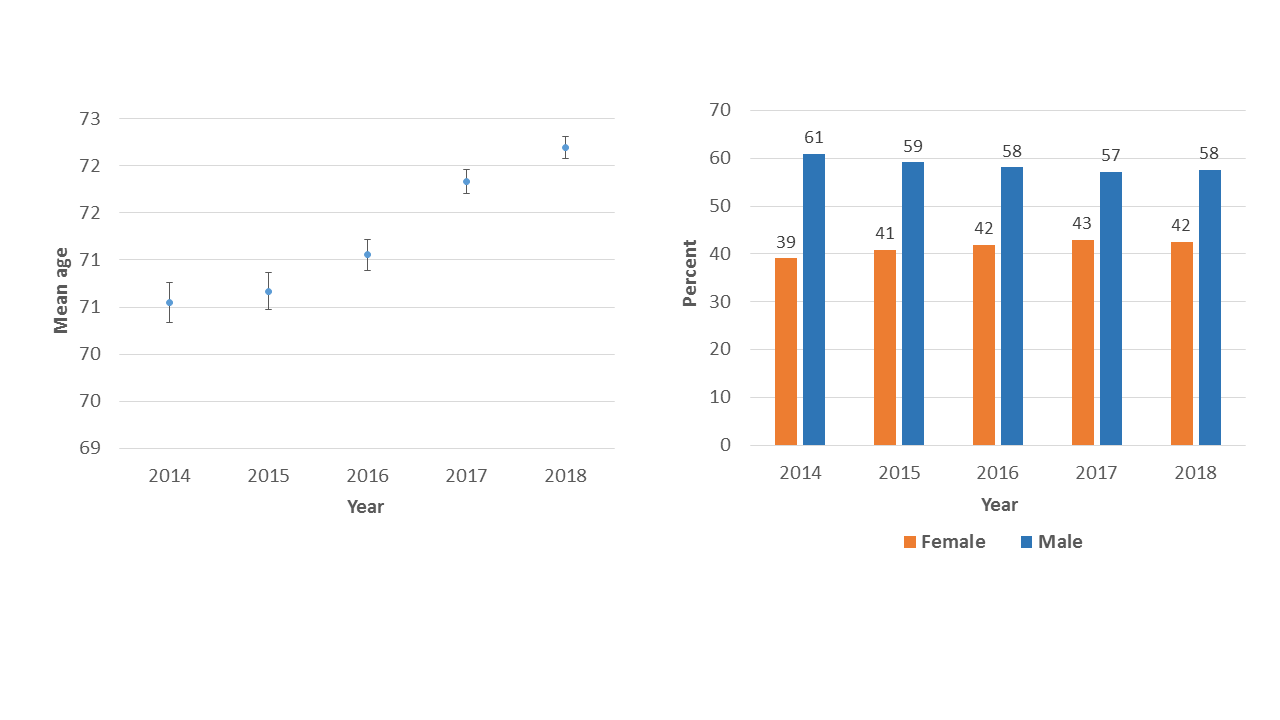

Supplement: Supplementary file 2 — Supplementary Fig. S1. Mean age and percentage female and male among new DOAC users (2014–2018). Left panel: displayed are mean age (points) and 95% confidence intervals (brackets) per study year. Right panel: percentage female (orange bars) and male (blue bars) per study year. DOAC direct oral anticoagulant [file 12471_2021_1612_MOESM2_ESM.tif]
